# Bridging access gaps experienced by the underserved: the need for healthcare providers to look within for answers

**DOI:** 10.1186/s12913-017-2756-4

**Published:** 2017-12-13

**Authors:** James K. Elrod, John L. Fortenberry

**Affiliations:** 1 Willis-Knighton Health System, 2600 Greenwood Road, Shreveport, LA 71103 USA; 2 LSU Shreveport, 1 University Place, Shreveport, LA 71115 USA

**Keywords:** Healthcare access, Community health, Medically underserved populations, Charity care

## Abstract

**Background:**

Health and medical providers dedicated to serving the poor face daunting challenges, with the most obvious one pertaining to the provision of services with little or no expectation of remuneration. This hardship often is overlooked by broad society as many view the delivery of healthcare services to indigent populations to be covered fully by government health insurance programs or other forms of public assistance. This, however, is only partially true and, even when reimbursements or similar payments are provided, they often fall short of covering the actual costs associated with rendering services.

**Discussion:**

With reimbursements from third parties often being unreliable, inadequate, and sometimes nonexistent, healthcare providers dedicated to serving poverty-stricken populations face quite a dilemma. As an institution which is devoted to addressing the disadvantaged, Willis-Knighton Health System has long sought remedies to bolster healthcare access for these vulnerable individuals. While public policy solutions ultimately are desired, historic and recent efforts continue to reveal fractures which in some cases have compelled providers to limit their exposure to indigent populations or withdraw from serving them altogether. Willis-Knighton Health System has addressed these challenges by operating as efficiently as possible, offering and successfully delivering a diverse service mix which permits a healthy margin that can support charitable care initiatives, and remaining steadfastly committed to shoring up indigent services in the community.

**Conclusions:**

Given the magnitude, scope, and expenditures associated with comprehensively addressing disadvantaged populations, public policy modifications appear to be the primary hope of remedying associated access gaps fully. Until effective measures are introduced, however, health and medical institutions dedicated to serving the indigent must look within for answers to associated challenges.

## Background

Poverty-stricken individuals face tremendous hardships in virtually every area of life, with access to healthcare services representing one of a seemingly endless array of trials and tribulations [1, 2]. Fortunately, many healthcare providers are dedicated to serving the poor, either exclusively, as in the case of indigent medical clinics, or as part of serving larger populations, as in the case of major medical centers which, as a component of a greater mission, supply health and medical care to those without the ability to pay for services. In fact, these charitably-minded care providers serve as healthcare lifelines for needy populations [3, 4]. Without these establishments, many would simply have to go without vital services, diminishing health and wellness and often exacerbating existing medical conditions, turning treatable health matters into chronic issues that can threaten life [5–7]. Often referred to as safety net providers because they effectively catch those who have fallen through gaps in the healthcare system [4, 8], these entities, too, face hardships which are different but no less daunting than those faced by the needy populations which they strive to serve.

The most obvious hardship faced by healthcare providers dedicated to serving the poor pertains to the provision of care with little or no expectation of remuneration. Regardless of population addressed, the delivery of healthcare services is an expensive undertaking [9]. Personnel, technology, equipment, space, and so on carry significant costs. Even when serving populations possessing excellent health insurance and high incomes to cover any out-of-pocket costs, the financial burdens of providing care are immense, making the prospect of little to no reimbursement for services a truly frightening proposition for most any healthcare establishment. Indeed, care that goes uncompensated can quickly drain even budgets which are well funded, threatening the viability of given healthcare institutions.

Hardships associated with uncompensated care often are overlooked by broad society as many view the delivery of healthcare services to indigent populations to be covered fully by government health insurance programs or other forms of public assistance. This, however, is only partially true and, even when reimbursements or similar payments are provided, they often fall short of covering the actual costs associated with rendering services. Government reimbursements for supplying care to the poverty stricken, in particular, are confounding for providers as these are subject to the public policy process and the degree of sentiment or lack thereof for addressing disadvantaged populations in the given political cycle. Even recent government initiatives to improve healthcare access, notably including mandatory health insurance, have not afforded full coverage of the populace, with the underprivileged being a most prominent casualty [10, 11]. If a provider is fortunate enough to receive reasonable reimbursements for caring for society’s most vulnerable today, such funding might not be available tomorrow, creating significant hesitance on the part of healthcare institutions to shore up access gaps in the communities they serve. Many, in fact, have sought to minimize their exposure to needy populations strategically and tactically, hastening an already burgeoning problem and effectively shutting out some individuals and communities from receipt of care altogether [12–14]. This, of course, places even greater burdens on those healthcare institutions which remain devoted to serving disadvantaged populations.

## Discussion

Over its many decades of service, Willis-Knighton Health System has faced and continues to face all of the challenges associated with delivering healthcare services to the underprivileged. Based in Shreveport, Louisiana and situated in the heart of an area known as the Ark-La-Tex where the states of Arkansas, Louisiana, and Texas converge, Willis-Knighton Health System holds market leadership in its served region where it delivers comprehensive health and wellness services through multiple hospitals, numerous general and specialty medical clinics, an all-inclusive retirement community, and more. Like many communities across America, especially those in the southeastern United States, significant poverty exists in Shreveport and the greater region. According to the United Way’s ALICE (Asset Limited, Income Constrained, Employed) Report which monitors those who are employed yet still unable to afford basic necessities and those suffering from poverty, 45% (114,912) of the households in northwest Louisiana are struggling to make ends meet. Of these households, 23% are ALICE and 22% are poverty stricken, making the region one of the poorest in the nation [15]. This requires concerted efforts on the part of healthcare providers to address the disadvantaged and Willis-Knighton Health System has done just that as part of its mission as a nongovernmental, not-for-profit institution [16]. The system, in fact, supplies more charitable care services than all other healthcare entities combined in its served market, leading many to consider it to be the safety net healthcare provider of the region, despite the state’s officially designated charity hospital for northwest Louisiana being located in close proximity.

Without Willis-Knighton Health System’s extensive efforts to serve the poor, the health status of the underprivileged in the marketplace would be abysmal, but all of these initiatives require vast resources in a policy environment which is not particularly friendly to elevating the status and stature of disadvantaged populations. Government programs designed to cover the healthcare expenses of the poor, such as Medicaid and the Louisiana Children’s Health Insurance Program (LaCHIP), typically supply reimbursements which fall far short of actual costs. Even the Patient Protection and Affordable Care Act, known more commonly as Obamacare, which promised among many other things to close access gaps for the disadvantaged, has not met intended goals [2, 10, 11]. Further, inequities exist between and among establishments regarding the reimbursements available to them for delivering uncompensated care, with Willis-Knighton Health System’s rates being a fraction of those granted to other healthcare institutions in the state as a result of disparate treatment emerging through the political process.

Across America, stories of providers taking steps to limit their exposure to the indigent due to public policy inadequacies have become commonplace. Often reported actions include the elimination of service lines which carry unfunded mandates that obligate providers to deliver care to anyone presenting, regardless of insurance status or ability to pay (e.g., emergency department services); the closure of campuses located in diverse neighborhoods and relocation of them to more prosperous areas populated by wealthier, better insured patients; and the exodus of medical practitioners from institutions dedicated to serving all to private, for-profit practices which serve only paying customers [12–14]. Regardless of whether one views these actions to be sinister, pragmatic, or somewhere in between, the disadvantaged are left with fewer health resources in their given neighborhoods. This, of course, places greater burdens on those healthcare institutions which remain devoted to serving the poor, as they must accommodate those who have been newly shut out of particular establishments and operations [12–14].

Willis-Knighton Health System indeed has faced temptations to restrict services through various means, but it has never succumbed to them. In perhaps the best example of this, many years ago, executives observed burgeoning growth in areas adjacent to west Shreveport, the community in which the institution’s sole campus at the time was located. Despite the attractiveness of abandoning its hospital and relocating to a more prosperous area, executives realized that by doing so, many patients of limited means living in the west Shreveport marketplace would no longer have a convenient option for receipt of services. As such, the decision was made to address these high-growth marketplaces with satellite facilities. The original west Shreveport campus would remain and receive extensive investments, effectively creating a main campus or hub for the growing system. This decision permitted growth, but did so in a manner that would not result in patient abandonment [17]. Another prominent example of Willis-Knighton Health System’s commitment to delivering charitable care is its establishment and operation of Project NeighborHealth, a network of indigent clinics situated within or near medically underserved communities, delivering health and wellness services to thousands of underprivileged residents. Further, uncompensated care of epic proportions is delivered in Willis-Knighton Health System’s emergency departments, urgent care clinics, and other locations, further illustrating efforts to support the indigent in the marketplace.

**Fig. 1:**
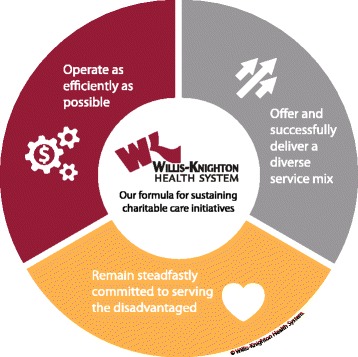
Willis-Knighton Health System’s formula for sustaining charitable care initiatives

But with public policy remedies falling short of reimbursement wants and needs, peer institutions increasingly abandoning the underserved, and patients who due to personal circumstances have no hope of being able to pay for healthcare services, what’s a charitably-minded healthcare provider to do to survive? Willis-Knighton Health System addressed this monumental dilemma by looking within for solutions, seeking pathways that permit continued efforts to serve the disadvantaged in the marketplace in a manner that does not threaten institutional viability or vitality. This particular approach revolves around three key areas: efficient operation, service line diversification, and commitment to serving the disadvantaged, with this array being illustrated in Fig. 1.

Willis-Knighton Health System possesses a lengthy history of operating in a manner characterized by the judicious use of resources. In fact, the institution has been described as possessing a culture of efficiency. Initiatives that greatly improve operational economies are commonplace throughout the system. Spatial expansion needs, for example, historically have been addressed where possible by repurposing abandoned buildings, adapting them for second lives as medical institutions for a fraction of the cost of new construction. Willis-Knighton Health System’s Project NeighborHealth indigent clinic network benefited directly from this approach, as associated efficiencies permitted the establishment of medical clinics that otherwise would not have been possible [18]. Additionally, efficiencies are realized by Willis-Knighton Health System’s use of the hub-and-spoke organization design which concentrates resources at its main campus or hub and places a more economical array of services at satellite campuses or spokes [17]. This particular method of structuring organizations is well known for its ability to deliver services more economically than other models, permitting Willis-Knighton Health System to direct the associated savings to other endeavors, including its charitable care initiatives. Many other aspects of operation feature equally efficient approaches, giving the system enhanced resources for aggressively serving disadvantaged populations.

Beyond efficient operation, service line diversification is critical, playing a key role in Willis-Knighton Health System’s successes addressing underserved populations. Quite obviously, diversification across multiple areas of health and wellness—something that major medical centers must do anyway—permits opportunities to fund charitable initiatives with gains afforded by prosperous service lines. This is a prudent practice regardless of mission, as reimbursement rates are not static and inflows received for one service line today can be restricted tomorrow, but it is especially vital for those engaged in addressing the disadvantaged, as uncompensated or poorly compensated care represents an absolute drain that cannot be supported over time without revenues achieved elsewhere. Inflows from prosperous service lines, especially when operations have been engineered for efficiency, supply lifeblood resources for essential, but unprofitable services which could not be offered otherwise. Without significant diversification and equivalent successes across multiple service lines, Willis-Knighton Health System’s altruistic endeavors would be vastly diminished.

A final component viewed by Willis-Knighton Health System to be critical for successful indigent care endeavors pertains to dedication. As noted earlier, some providers have taken steps to reduce or eliminate their exposure to disadvantaged populations. From Willis-Knighton Health System’s perspective, such occurrences simply are the result of these entities not possessing the required level of dedication needed to stay the course. Indeed, establishments must be steadfastly committed to addressing the medically underserved, understanding that hardships will be incurred by such, but also realizing that by taking prudent actions, abandonment of the less fortunate is not necessary. Healthcare entities truly dedicated to serving disadvantaged populations will find ways, as impossible as they may seem, to ensure the continuation of their altruistic missions. Willis-Knighton Health System has done just that, courtesy of its total and complete dedication to serving the less fortunate, a key ingredient in successful indigent care pursuits.

## Conclusions

Willis-Knighton Health System’s approach for addressing the underserved resulted from environmental conditions which required it to look within for answers to ensure that the less fortunate in the marketplace remained in excellent care. In doing so, the institution was able to combine several elements of its operational philosophy to reduce the impact of public policy inadequacies and their associated ramifications. Willis-Knighton Health System’s intensive efforts have yielded robust indigent care services which have improved the state of community health. Each institution seeking to continue providing healthcare services for the disadvantaged is encouraged to follow Willis-Knighton Health System’s method and look within for tools and techniques that will permit perpetuation of the noble mission of serving the less fortunate. It is hoped that the approach portrayed in this article will be of use to charitably-minded healthcare institutions far and wide, perhaps giving others a prudent course of action or at least stimulating related ideas that can ensure continuity of service. The disadvantaged need healthcare services right now. Waiting for public policy remedies which may never arrive is not a viable option, nor is it prudent to hope that establishments which have abandoned indigent populations will someday welcome them back into their institutions. Healthcare entities dedicated to serving the poor must look within for answers to associated challenges.
